# ABI4 and its role in chloroplast retrograde communication

**DOI:** 10.3389/fpls.2012.00304

**Published:** 2013-01-10

**Authors:** Patricia León, Josefat Gregorio, Elizabeth Cordoba

**Affiliations:** Departamento de Biología Molecular de Plantas, Instituto de Biotecnología, Universidad Nacional Autónoma de MéxicoCuernavaca, Morelos, México

**Keywords:** retrograde communication, ABI4, plastids, chloroplast, nuclear photosynthetic genes, signaling

## Abstract

The acquisition of plastids is a landmark event in plant evolution. The proper functionality of these organelles depends on strict and continuous communication between the plastids and the nucleus to precisely adjust gene expression in response to the organelle’s requirements. Signals originating from the plastids impact the expression of a variety of nuclear genes, and this retrograde communication is essential to couple the nuclear expression of plastid-localized products with organelle gene expression and, ultimately, functionality. Major advances have been made in this field over the past few years with the characterization of independent retrograde signaling pathways and the identification of some of their components. One such factor is the nuclear transcriptional factor ABI4 (ABA-INSENTIVE 4). ABI4, together with the plastid PPR GUN1 protein, has been proposed to function as a node of convergence for multiple plastid retrograde signaling pathways. ABI4 is conserved among plants and also plays important roles in various critical developmental and metabolic processes. ABI4 is a versatile regulator that positively and negatively modulates the expression of many genes, including other transcriptional factors. However, its mode of action during plastid retrograde signaling is not fully understood. In this review, we describe the current evidence that supports the participation of ABI4 in different retrograde communication pathways. ABI4 is regulated at the transcriptional and post-transcriptional level. A known regulator of ABI4 includes the PTM transcription factor, which moves from the chloroplast to the nucleus. This transcription factor is a candidate for the transmission of retrograde signals between the plastid and ABI4.

The endosymbiotic acquisition of the chloroplast and mitochondria by eukaryotic cells are two of the most important events in the history of life on Earth (Margulis, 1971). Chloroplasts provide plants with autotrophic capacity, and their byproducts are the source of the majority of the carbon skeletons of all living organisms. Today, chloroplasts host not only the photosynthetic pathway but also other essential metabolic pathways, many of which are readily traced to prokaryotic ancestors (Gould et al., 2008). These include the synthesis of amino acids, fatty acids, and vitamins. Additionally, hormones and an enormous number of secondary metabolites, many of them important to humans, are also synthesized in plastids. Coevolution between the original endosymbiont and the plant cell has resulted in a heterogenous family of plastid types, each with specialized functions and all essential for plant survival (Neuhaus and Emes, 2000; Vothknecht and Westhoff, 2001).

The plastid genome encodes around 100 genes. Therefore, more than 95% of the proteins required for plastid function are encoded in the nucleus, and the corresponding proteins are imported into the organelle post-translationally. This latter step is achieved through a transit peptide present at the N-terminus that suffices for import into the organelles, found in the majority of nuclear-encoded plastid proteins (NEPPs; Li and Chiu, 2010). However, with the massive transfer of genes to the nucleus, the organelles lost autonomy and became dependent on the host. The nucleus gained control over the expression of the transferred genes and consequently over the plastid function and development in what is known as anterograde regulation.

*In silico* predictions based on the presence of putative transit peptides yield estimates of 2500–4500 NEPPs (Richly and Leister, 2004). Although the location of many of these proteins has not been experimentally proven, large-scale proteomics experiments http://ppdb.tc.cornell.edu/) corroborate the complexity of the plastid proteome (Zybailov et al., 2008; Joyard et al., 2009). Moreover, these analyses elucidated proteomic differences between plastid types and within developmental stages, demonstrating that plastid protein composition is both complex and dynamic (Zybailov et al., 2008). A significant proportion of the proteins imported into plastids form macromolecular complexes (Olinares et al., 2010). Examples include complexes of the photosynthetic apparatus as well as those involved in organelle maintenance, such as ribosomes and DNA or RNA polymerases (Kovacs-Bogdan et al., 2010; Olinares et al., 2011). In many cases, these complexes consist of subunits that are encoded in both the nucleus and the plastid genomes, with functions that depend on proper stoichiometry. The process is further complicated by the requirement of some of these complexes for specific cofactors and the reorganization and replacement or repair of specific subunits under certain conditions (Rochaix, 2011; Janska et al., 2012). While it has been established that post-translational regulatory processes play an important role in this coordination (Rochaix, 2011), it is also clear that coordination between the nucleus and the organelle at the level of gene expression is an essential element to ensuring proper organelle functionality. As was already mentioned, the nucleus regulates plastid gene expression through regulating NEPPs (Woodson and Chory, 2008; Stern et al., 2010). Nonetheless, retrograde mechanisms that permit the mitochondria and plastids to transmit their developmental and metabolic status to the nucleus have evolved, resulting in the modulation of nuclear gene expression in response to the needs of the organelle.

## RETROGRADE REGULATION IS AN ESSENTIAL MECHANISM FOR ORGANELLE FUNCTIONALITY

Since the isolation of the *albostrians* (*Hordeum vulgare* cv. Haisa) mutant more than 30 years ago (Hedtke et al., 1999), data have accumulated that demonstrate the existence of multiple plastidial retrograde signaling pathways in response to specific developmental and metabolic cues of the plastids. In all cases, it is assumed that a signal(s) must exit the plastid to transmit information directly or indirectly to components in the nucleus to fine-tune nuclear gene expression. Different pathways have been defined based primarily on the regulated nuclear genes and the participating factors. Most of our present knowledge is based on studies from *Arabidopsis thaliana* using inhibitors and mutants that impair organelle development (Rapp and Mullet, 1991; Hedtke et al., 1999; Sullivan and Gray, 1999; Pesaresi et al., 2001; Nott et al., 2006; Woodson and Chory, 2008). In recent years, the diversity and complexity of signaling pathways have necessitated more dynamic consideration to enhance our understanding of these events (Leister, 2012). Advances in this field have been sizeable and are covered in detail in various reviews (Mullineaux and Karpinski, 2002; Nott et al., 2006; Pesaresi et al., 2007; Pogson et al., 2008; Woodson and Chory, 2008; Chan et al., 2010; Galvez-Valdivieso and Mullineaux, 2010; Inaba et al., 2011). The purpose of this review is to describe the recent identification of the factors that are recognized as important elements in regulating the expression of NEPPs in response to retrograde signals.

A common effect of all retrograde signaling pathways is the alteration of nuclear gene expression, and the available evidence indicates that these changes are, in most cases, at the level of transcription (Richly et al., 2003; Leister et al., 2011). An exception to this is a recently described pathway, referred to as PAP (3′-phosphoadenosine 5′-phosphate). PAP itself moves to the nucleus, acting as a true signal, and increases gene expression by inhibiting the activity of the nuclear 5′ to 3′ exoribonucleases (XRN2 and XRN3), which act as negative regulators of the high light and drought stress responses (Dichtl et al., 1997; Estavillo et al., 2011). This example illustrates the possibility that post-transcriptional regulators such as ribonucleases also participate in the reprogramming of nuclear expression caused by retrograde signals (Estavillo et al., 2011).

Early analyses of the retrograde chloroplast signaling pathways primarily monitored a few particular genes like *LHCB* and *RBCS* (Bradbeer et al., 1979; López-Juez, 2009). Identification of additional targets has been used to define distinctive retrograde pathways, their potential interactions and overlapping components, and the identification of candidate signaling molecules responsible for the changes in gene expression (Koussevitzky et al., 2007; Brautigam et al., 2009; Leister et al., 2011). Initial genomic studies from wild-type plants and mutants with altered retrograde signal responses, such as *genomes uncoupled* (*gun*), which no longer represses NEPPs in the presence of norflurazon, an inhibitor of carotenoid biosynthesis (Susek et al., 1993), demonstrated the coordination of many chloroplast genes following a two-state switch behavior (on or off). Based on these data, it was proposed that master switches existed for several retrograde responses (Richly et al., 2003). *Cis*-acting elements involved in abscisic acid (ABA) responses that are over-represented in the upstream regions of the *gun*-affected genes and known putative regulatory factors that may interact with these sequences have been identified (Niu et al., 2002; Koussevitzky et al., 2007). One such regulator is the ABA-INSENTIVE 4 (ABI4) transcription factor, which was predicted to be important in plastidic retrograde signaling pathways (Koussevitzky et al., 2007).

## ABI4 IS A VERSATILE REGULATOR FOR MULTIPLE SIGNALING RESPONSES

The ABI4 protein belongs to the DREB A3 subgroup of a large family of plant-specific transcription factors known as AP2/EREBP (Sakuma et al., 2002). The *A. thaliana* genome encodes 147 AP2/EREBP members, and many of them are of particular interest because they are implicated in many signaling processes, including biotic and abiotic stress responses (Mizoi et al., 2012). In spite of the similarity between the AP2 DNA-binding domain with other members of the DREB A subgroup, ABI4 stands out as a unique member in the A3 clade based on its sequence (Dietz et al., 2010). Orthologs of ABI4 have been reported in maize and rice (Niu et al., 2002), and homologous sequences are found in many plant species, indicating that this factor is conserved in most plants. In *A. thaliana*, ABI4 is a unique gene, and this also appears to be true for other plants such as maize and rice.

**FIGURE 1:**
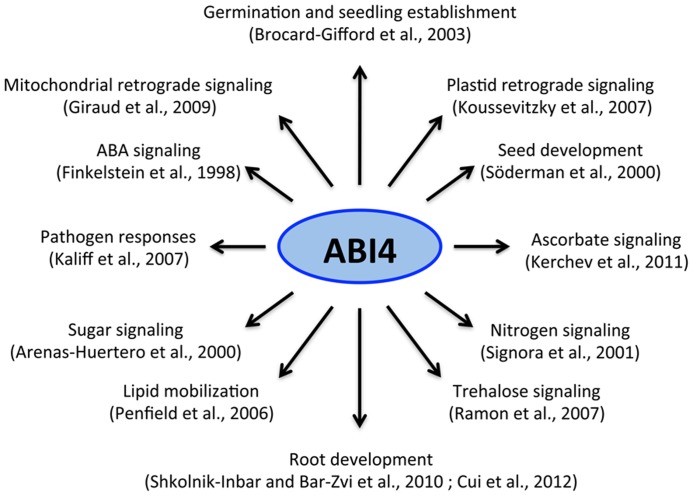
**ABI4 and its role in plant growth and development**. ABI4 acts as a node of convergence of multiple signals that regulate different processes during plant development.

**FIGURE 2:**
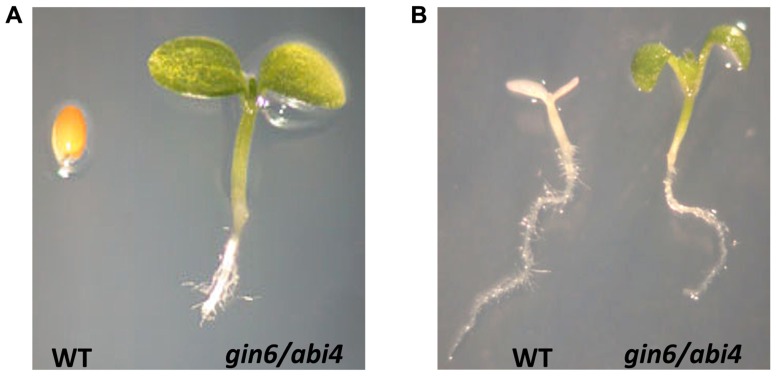
**ABI4 is required for ABA and sugar signaling**. The *abi4*/*gin6* mutant plant is ABA-insensitive (*abi*) and glucose-insensitive (*gin*). *A. thaliana* wild-type plants are unable to grow in 5 μM of ABA, whereas the *abi4* mutant plant develops normally **(A)**. In media containing 6.5% glucose (Glc), the development of wild-type plants arrests, and they do not accumulate chlorophyll in contrast to the *abi4*/*gin6* mutant, which continues growing and displays a green phenotype and cotyledon expansion **(B)**.

Over the past decade, ABI4 has emerged as a central player in many signaling processes during plant development (**Figure 1**). The isolation of ABI4 in a screen for ABA-insensitive (*abi*) mutants was the first evidence linking this factor with ABA signaling (Finkelstein et al., 1998). Unlike wild-type plants, *abi4* mutants display resistance to the ABA inhibitory effect during germination and seedling and chloroplast development (**Figure 2A**). Additional *abi4* alleles were isolated in several screenings for mutants with altered responses to sugars (Arenas-Huertero et al., 2000; Huijser et al., 2000; Laby et al., 2001; Rook et al., 2001). The presence of high sugar levels triggers post-germinative seedling arrest (chloroplast and leaf development) in wild-type plants, but not in the *abi4* mutant (**Figure 2B**). ABI4 and hormones such as ABA and ethylene play an essential role in sugar perception during early seedling development (León and Sheen, 2003; Rolland et al., 2006; Rook et al., 2006a) and in the primary root (Cui et al., 2012). The developmental arrest in response to sugars only occurs during early developmental stages, similar to some of the retrograde chloroplast signaling responses (Dekkers et al., 2008). Sugars and ABA have been proposed to act as important signals during early development by directly controlling germination, photo-autotrophic development, the expression of photosynthetic genes (Baier and Dietz, 2005; Katagiri et al., 2005; Cottage et al., 2010; Lee et al., 2012) and even the initiation of plastid retrograde signaling (Koussevitzky et al., 2007). ABI4 also plays an important role in other plant functions including nitrogen signaling, lipid mobilization in the embryo, root growth, lateral root inhibition, and pathogen resistance (Signora et al., 2001; Jakab et al., 2005; Penfield et al., 2006; Kaliff et al., 2007; Shkolnik-Inbar and Bar-Zvi, 2010; Kerchev et al., 2011; Cui et al., 2012). Recent studies demonstrated that ABI4 is also required for redox responses in ascorbate-mediated signaling (Kerchev et al., 2011). These studies provided evidence of a close interaction between the redox and sugar signaling pathways (Foyer et al., 2012), which further supports the prominent role of ABI4 as a point of convergence for various signaling pathways (**Figure 1**).

For the purpose of this review, it is particularly interesting that the ABI4 transcription factor has been found to be important in chloroplast retrograde signaling pathways (see below) and in mitochondrial retrograde communication. The induction of the nuclear-encoded alternative oxidase gene (*AOX*) in response to defects in electron transport chain inhibition by rotenone has been commonly used as a marker for mitochondrial retrograde signaling responses (Rhoads et al., 2006; Rhoads and Subbaiah, 2007). This induction is abolished in the *abi4* mutant during rotenone challenge (Giraud et al., 2009). ABI4 was demonstrated to bind directly to upstream elements of the *AOX* gene. These data indicate that ABI4 also plays a direct role in mitochondrial retrograde signaling in response to defects in the electron transport chain.

## ABI4 AND ITS FUNCTION IN PLASTID RETROGRADE SIGNALING DURING PLASTID DEVELOPMENT

Based on their functional implications, plastid retrograde signals have been divided into those related to plastid development and those involved in operational fine-tuning in response to environmental or metabolic fluctuations (Pogson et al., 2008). This classification is useful, but it is not absolute because diverse lines of evidence demonstrate that several of these pathways are interconnected and share various components. The transition from proplastid to chloroplast is probably one of the most critical moments during seedling establishment because autotrophic capabilities depend on this process. During this time, and in response to defects in the developmental process, at least three different pathways have been reported that produce particular signal(s) and regulate NEPPs involved in the photosynthetic pathway and in plastid maintenance. These pathways are known as the plastid gene expression (PGE), tetrapyrrole, and plastid protein import (Nott et al., 2006; Woodson and Chory, 2008; Inaba et al., 2011).

**FIGURE 3:**
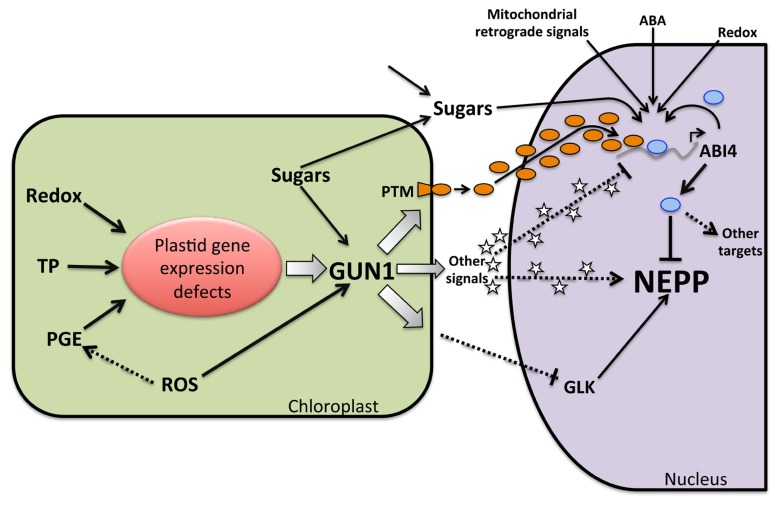
**Model of GUN1 and ABI4 function as integrators of NEPP regulation from several retrograde signaling pathways**. Multiple conditions that alter plastid functionality, such as inhibition of plastid transcription or translation (PGE), tetrapyrrole biosynthesis inhibition (TB), altered redox status (Redox), and ROS production, result in defects in plastid gene expression. This model demonstrates how defects in plastid gene expression are capable of producing multiple signals, in close cooperation with GUN1, which acts as a convergent node. These signals can be positive or negative, resulting in the processing of the PTM factor in the plastid envelope and allowing its translocation to the nucleus (black circles), for example. In parallel, positive signals like heme (stars) generated by the plastid under normal conditions will decrease (represented by a smaller arrow). These events will lead to an increase in the level of the transcriptional regulator ABI4 (blue ovals) as a result of the induction of its transcription by factors such as PTM (orange ovals) or by the decrease of positive regulators. Elevated levels of ABI4 could also be the result of a reduction of its degradation. ABI4 acts as a repressor of photosynthetic nuclear genes and as an activator of other genes, such as those related to carbon catabolism. Positive signals such as heme (stars) could act by inhibiting the ABI4 expression or activating NEPPs. Defects in plastid gene expression mediated by GUN1 could modulate the levels of other factors such as GLKs that act as positive regulators of NEPPs. The final outcome in gene expression will depend on the levels of multiple positive and negative signals. ABI4 also responds to other signals such as the level of sugars produced inside the chloroplast or imported into the cell, ABA, redox status and retrograde signals from the mitochondria, which are all relevant for plastid function and modulate the NEPPs.

Independent analyses have reported the participation of ABI4 in the PGE and tetrapyrrole retrograde signaling pathways (**Figure 3**). PGE appears to be important in coordinating the NEPPs required for different plastid types in early development (Kanervo et al., 2008). In response to defects in plastid gene expression or translation, a down-regulation of the expression of multiple NEPPs such as *LHCB* was observed (Pesaresi et al., 2006; Koussevitzky et al., 2007). The PGE pathway has primarily been characterized with the use of inhibitors that impair organelle transcription (Rapp and Mullet, 1991) or translation, such as lincomycin (Sullivan and Gray, 1999). This pathway has also been analyzed with mutants that affect early organelle development (Hedtke et al., 1999; Pesaresi et al., 2001). Although various studies have reported that the PGE pathway functions predominantly during the first days after germination (Sullivan and Gray, 1999), the characterization of the effect of the *prors1* mutant in chloroplast translation suggests that this regulation persists in mature leaf tissues (Pesaresi et al., 2006). The signal(s) that initiates this pathway is still unknown, but recent data showed that changes in plastid gene expression in response to redox changes, could potentially trigger the PGE pathway. This establishes that the expression of the NEPPs is subject to physiological regulation by the redox status of the organelle. The recent characterization of the plastid redox-insensitive *prin2* mutant in *A. thaliana* supports this regulatory mechanism (Kindgren et al., 2012). PRIN2 is a plastid protein that localizes to the plastid transcriptionally active chromosome (pTAC) complex, and it enhances plastid transcription mediated by the plastid-encoded RNA polymerase (PEP). Consequently, in the *prin2* mutant, expression of plastid-encoded genes transcribed by the PEP is low, showing a similar profile to that observed in mutants with impaired PEP activity (Chateigner-Boutin et al., 2008; Arsova et al., 2010). Apparently, the low expression levels of plastid genes in the *prin2* mutant disrupt positive signals or induce negative signals that decrease the expression of NEPPs such as *LHCB* (**Figure 3**). Collectively, all these findings support the hypothesis that plastid gene expression mediated by PEP acts as a central integrator to initiate PGE retrograde signaling in response to developmental and metabolic cues.

One factor required in PGE signaling is GUN1, a plastid protein and member of the pentatricopeptide repeat (PPR) family. The *gun1* mutant is defective in the repression of NEPPs in response to lincomycin, and consequentially, *LHCB* expression is high in this mutant, a notable characteristic of the *gun* phenotype (Koussevitzky et al., 2007; Cottage et al., 2010; Sun et al., 2011). Interestingly, a weak *gun* phenotype was also observed in the *abi4* mutant in the presence of lincomycin, suggesting that this transcription factor plays a role in the PGE-dependent signaling pathway (**Figure 3**). Accordingly, overexpression of ABI4 suppresses the *gun* phenotype in the presence of lincomycin (Koussevitzky et al., 2007). However, the *gun* phenotype of the ABI4 mutant appears to be weaker than other *gun* mutants, and may be dependent on the high light conditions used during its analysis (Voigt et al., 2010; Kerchev et al., 2011), suggesting that other transcriptional regulators may also participate in the repression of NEPPs mediated by PGE (**Figure 3**).

Another retrograde signaling pathway involved in plastid development, which is probably the most extensively studied, is commonly referred to as the tetrapyrrole pathway. This pathway is associated with defects in the biosynthesis of chlorophyll (Nott et al., 2006; Woodson and Chory, 2008). In higher plants, the disruption of chloroplast development in response to photodamage caused by inhibitors of carotenoid biosynthesis such as norflurazon is associated with major changes in NEPP expression and the accumulation of intermediate tetrapyrrole compounds (Strand et al., 2003). Components of this signaling pathway were identified by isolating different *gun* mutants that no longer repressed NEPPs in the presence of norflurazon (Susek et al., 1993). Several of the characterized *gun* mutants affected different steps in porphyrin biosynthesis (Nott et al., 2006). These data led to the postulation that the tetrapyrrole intermediate Mg ProtoIX can be actively transported from the chloroplast to the cytosol and acts as a plastid signal (Mochizuki et al., 2001; Beck, 2005; Ankele et al., 2007). However, the role of the Mg ProtoIX as a signaling agent in this pathway has been questioned (Mochizuki et al., 2008; Moulin et al., 2008), and other molecules such as reactive oxygen species (ROS) and heme have been proposed to function as alternative signals (Mochizuki et al., 2008; Moulin et al., 2008; Woodson et al., 2011). The recent characterization of a new *gun* mutant (*gun6*) showed that excess accumulation of heme acts as an activator of the expression of photosynthetic genes. The role of heme as a signal that positively regulates NEPPs is particularly attractive not only because this molecule is known to be actively transported out of the plastids (Thomas and Weinstein, 1990) but also because heme functions as a signal in other organisms (Mense and Zhang, 2006; von Gromoff et al., 2008).

GUN1 is an essential factor in this signaling pathway and has been proposed to function as a node of convergence in the tetrapyrrole and PGE retrograde signaling pathways (Koussevitzky et al., 2007; Kakizaki et al., 2009). The function of the PPR protein GUN1 is still unclear, but very intriguing. PPR proteins have diverse functions, primarily related to organellar RNA metabolism (Schmitz-Linneweber and Small, 2008). Most of these proteins have specific RNA binding activity. Therefore, it has been speculated that a possible function for GUN1 is that it binds to a particular chloroplast transcript(s) that is essential for the generation of a chloroplast retrograde signal(s) (Woodson et al., 2012). GUN1 colocalizes with other PPR proteins such as pTAC2, a component of the pTAC that is involved in both transcription and post-transcriptional plastid processes (Ding et al., 2006; Pfalz et al., 2006). In addition to the PPR motifs, GUN1 and pTAC2, together with other five PPR proteins, share a “short mutS-related” domain that has been shown to have DNA-binding functions (Koussevitzky et al., 2007), indicating that these molecules maybe involved in the regulation of plastid transcription under certain conditions. Recently, Woodson et al. (2012) demonstrated that plastid transcription is a key element for the generation of at least two independent retrograde signals. Mutations in the sigma factors *sig2* and *sig6*, which are part of the PEP, result in the low expression of dozens of plastid-encoded and nuclear-encoded photosynthetic genes. Transcriptomic analysis demonstrated similarities in the expression pattern of these mutants compared to those of lincomycin- and norflurazon-treated plants. A key finding was that GUN1 is responsible for the defects observed in *sig2* and *sig6* mutants. Among the plastid genes with altered transcription levels in these *sig* mutants is tRNA^Glu^, a key substrate of tetrapyrrole synthesis (Schon et al., 1986; Hanaoka et al., 2005). Accordingly, in *sig2* and *sig6* mutants, the levels of tetrapyrroles and heme are low. Increasing the levels of heme partially suppressed the expression defects only in the *sig2* mutant, supporting the role of this molecule as a positive regulator of nuclear gene expression (Woodson et al., 2012). These results add to the evidence of the role that plastid transcription plays in the generation of different signal(s) for PGE and tetrapyrrole retrograde pathways. One of such signals apparently originates directly from the transcription defects of the plastid genome. The other signal is related to the low levels of heme that result from the *sig* mutation. These results also position GUN1 as a strategic transmitter of these signals through a post-transcriptional regulatory mechanism by regulating the stability or the of plastid transcripts such as tRNA^Glu^ (Woodson et al., 2012). Several lines of evidence support the idea that ABI4 is also important in the transmission of tetrapyrrole pathway signals from the chloroplast to the nucleus. As in the *gun* mutants, the repression of the *LHCB* transcript in the *abi4* mutant is attenuated to approximately one-third that of the control plants after norflurazon treatment. Moreover, *gun1* has been shown to be epistatic to *abi4* because the expression of *LHCB* in the *gun1abi4* double mutant resembles that found in *gun1*. Similar to the PGE pathway, overexpression of ABI4 is capable of suppressing the misregulation of the *LHCB* gene observed in the *gun1* mutant (Koussevitzky et al., 2007). Finally, recent data showed that the expression of *ABI4* is induced by both lincomycin and norflurazon treatment (Sun et al., 2011), supporting a role for ABI4 in the retrograde responses of the PGE and tetrapyrrole pathways (**Figure 3**). However as previously mentioned, the fact that the expression level of genes such as *LHCB* in the *gun1* and *abi4* mutants never reaches that observed in control plants without treatment also suggests that additional factors or a more complex mechanism participate in NEPP repression caused by the disruption of chloroplast development.

## ABI4 AND THE RETROGRADE OPERATIONAL CONTROL PATHWAYS

Changes in chloroplast function in response to high light or environmental fluctuations trigger different retrograde signaling responses classified as operational control pathways (Pogson et al., 2008). These pathways can adjust to changes in organelle status or initiate stress responses after organelle damage. Several of these pathways are triggered by specific ROS or by the redox status inherent to photosynthetic electron transfer (PET) and may be mostly related to chloroplasts (Foyer and Noctor, 2009; Galvez-Valdivieso and Mullineaux, 2010). Other pathways respond to the production of particular metabolites such as PAP (Estavillo et al., 2011) and MecPP (methylerythritol cyclodiphosphate; Xiao et al., 2012), which can potentially be produced by numerous plastid types.

Several lines of evidence support the concept that specific ROS species trigger independent signaling pathways, and these have been extensively reviewed (Pogson et al., 2008; Foyer and Noctor, 2009; Galvez-Valdivieso and Mullineaux, 2010; Mittler et al., 2011). The presence of high light levels damages chloroplasts and results in high levels of singlet oxygen (^1^O_2_), leading to a rapid inhibition of plant growth, the induction of plant cell death and the differential regulation of specific sets of nuclear genes (op den Camp et al., 2003). The characterization of the *flu* mutants that hyperaccumulate photosensitive compounds that generate ^1^O_2_ supported the hypothesis that this ROS elicits a specific retrograde signaling pathway that is active during embryogenesis and impacts on plastid differentiation during germination (op den Camp et al., 2003; Lee et al., 2007; Kim et al., 2009). Recent data indicate that the ^1^O_2_ pathway is also active under moderate light stress conditions, causing limited cell death that may be part of the acclimation response in plants that enhances stress resistance (Kim et al., 2012). Interestingly, the production of high ^1^O_2_ levels has also been reported during norflurazon and lincomycin treatments, and this molecule has been considered to be a putative signal for the modulation of NEPPs in response to these inhibitors. As previously described, this regulation is no longer observed in mutants of GUN1 and ABI4, supporting a link between ^1^O_2_ and these transcription factors (Moulin et al., 2008).

A second class of ROS that has been shown to elicit specific gene expression responses is hydrogen peroxide (H_2_O_2_; Cruz de Carvalho, 2008). Various stresses, including photoreduction of O_2_ at photosystem I, high light, and herbicides such as paraquat, induce H_2_O_2_ production. Under these conditions, the expression of many genes related to biotic and abiotic stresses, such as ascorbate peroxidase and the nuclear transcription factors ZAT10 and ZAT12, are induced (Rossel et al., 2007; Maruta et al., 2012). Activation of a specific MAP protein kinase cascade is required for signal transduction (Nakagami et al., 2006; Pitzschke and Hirt, 2009). Mutations in the *GUN1* and *ABI4* genes also affect the induction of *ZAT10* and *ZAT12*. These findings have been taken as evidence of the participation of ABI4 and GUN1 in ROS-mediated retrograde pathways (Koussevitzky et al., 2007; Moulin et al., 2008). A key question that is still unanswered is which specific ROS molecule “converges” at GUN1 and consequently at ABI4, given that high light treatment can induce the production of free radicals (superoxide anion, O_2_^-^ and hydroxyl radical, ^-^OH) and non-radical molecules (^1^O_2_, and H_2_O_2_).

Subtle changes in temperature, light quality, and intensity have tremendous effects on the chloroplasts’ redox state. These changes are closely linked to the function of PET. Changes in the redox status of the plastoquinone (PQ) pool and thioredoxin proteins permit long-term adjustments that result in changes in the accumulation of photosynthetic components such as the NEPPs (Kleine et al., 2007; Wagner et al., 2008; Brautigam et al., 2009). Thioredoxins are also major redox transmitters that modulate the activity of many plastidial enzymes and regulatory proteins (Arsova et al., 2010; Pesaresi et al., 2010; Dietz and Pfannschmidt, 2011). The characterization of a novel plastidial thioredoxin isoform (TRX z) links these proteins to plastid gene expression and possibly also with retrograde signaling (Arsova et al., 2010). TRX z regulates the activity of PEP through the action of two nuclear-encoded kinases related to fructokinases (FLN1 and FLN2). These changes in plastid gene expression in response to the redox state could potentially trigger PGE-mediated retrograde signaling during early seedling development, establishing physiological regulation of NEPPs by the redox status of the organelle. As mentioned above, the low plastid gene expression levels in the *prin2* mutant disrupt positive signals or induce negative signals that decrease the expression of NEPPs (Kindgren et al., 2012). Collectively, these findings support the concept that plastid gene expression mediated by PEP functions as a central integrator to initiate retrograde signaling in response to developmental and metabolic cues. At this point, it is still an open question whether this redox-mediated regulation depends on GUN1 and ABI4.

## ABI4 AS A REGULATOR OF NUCLEAR GENE EXPRESSION

Analysis of the expression patterns of the *abi4* mutant (Koussevitzky et al., 2007; Kerchev et al., 2012) or transgenic plants overexpressing ABI4 (Reeves et al., 2011) has demonstrated that ABI4 modulates the expression of a significant number of genes. Among its potential targets are genes involved in seed development and maturation, genes involved in metabolism, genes related to stress and defense and, finally, several transcription factors. These analyses have also revealed interesting overlaps in the expression profiles of other mutants including *gun1* (Koussevitzky et al., 2007) and the ascorbate-deficient mutants *vtc1* and *vtc2* (Kerchev et al., 2012).

**FIGURE 4:**
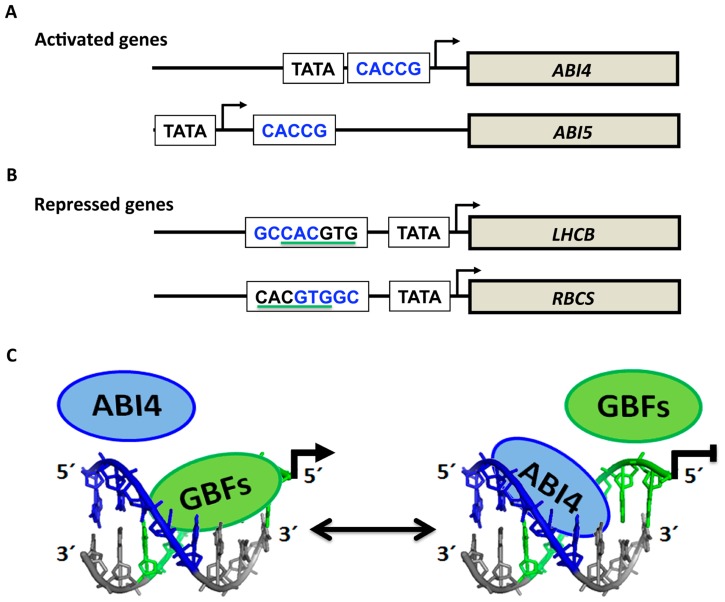
**Target genes of ABI4**. **(A)** Genes activated by ABI4 generally contain the CE1-like sequence (CACCG; in blue) identified by Niu et al. (2002). In contrast, repressed genes contain the CE1-like element in the reverse orientation (GCCAC) and overlap with the G-box element (underlined in green; **B**). The proposed model of repression by ABI4 is depicted in **(C)**. Competition for the binding site between ABI4 and GBF (G-box binding factor) results in non-activation of GBF target genes represented by the line in **(C)**. For ease of interpretation, the G-box is shown on the complementary-sense strand (green), whereas the CE1-like element (in blue) is depicted on the direct sense-strand. The GCCACGTG sequence was modeled by 3D-DART (Van Dijk and Bonvin, 2009) and visualized with PyMOL (http://www.pymol.org/).

In *in vitro* and transient assays, ABI4 has been shown to bind to the CE1-like element CACCG, which is present in the promoters of its putative target genes in *A. thaliana* (Finkelstein et al., 1998; Niu et al., 2002; Sakuma et al., 2002; Acevedo-Hernandez et al., 2005; Koussevitzky et al., 2007; Bossi et al., 2009; Giraud et al., 2009; Yang et al., 2011; Lee et al., 2012) and maize (Hu et al., 2012). Binding to this sequence results in the activation or repression of target genes, including ABI4 itself (Acevedo-Hernandez et al., 2005; Rook et al., 2006b; Bossi et al., 2009). The dual function of this factor as a repressor or an activator apparently depends on the context of its binding site in conjunction with the binding site of essential activators (**Figures 4A**,**B**). In several repressed genes from different species such as *LHCB* and *RBCS* (Figure 4B), the ABI4 binding site overlaps with a G-box element (CACGTG), which is essential for high levels of expression in many light-regulated genes (Acevedo-Hernandez et al., 2005; Koussevitzky et al., 2007). In these two cases, the CE1-like element is present in a different orientation than in the activated genes (**Figure 4A**). It has been proposed that ABI4 competition for binding with activators such as G-box binding factors (GBFs) results in ABI4-mediated reduction of gene expression (**Figure 4C**; Acevedo-Hernandez et al., 2005; Rook et al., 2006b). Consistent with this arrangement for the repressed genes, most of the ABI4-specific activated targets identified by Reeves et al. (2011) do not contain the CE1-like element (GCCAC) that overlapped with a putative G-box in either orientation. Moreover, the GCCACGTG or CACGTGGC sequences in which a putative CE1-like element overlaps with a putative G-box, have been found enriched in promoters of photosyntheyic genes, which could be potential targets for repression by ABI4 (Joung et al., 2009). Finally, studies based on transcriptomic analysis of the *abi4* mutants or plants ectopically expressing ABI4 support that ABI4 positively or negatively regulates the expression of a significant number of genes involved in photosynthesis, hormone signaling and defense, among others (Foyer et al., 2007; Koussevitzky et al., 2007; Reeves et al., 2011). From these analyses, it was also suggested that ABI4 might have additional alternative binding sites, including a shorter version of the CE1-like motif (CCAC) or a motif that overlaps with ABRE sequences (Koussevitzky et al., 2007; Reeves et al., 2011).

## ABI4, AN ELUSIVE REGULATORY FACTOR

Despite support for the role of ABI4 as an important regulator in many processes, the mechanism through which this factor integrates different signals remains unknown. An important limitation to experimental investigation of this protein is that the ABI4 protein has been extremely difficult to detect. Most of our present knowledge about ABI4 comes from its analysis at the transcriptional level. The expression pattern of *ABI4* has been inferred mostly following the activity of the GUS marker fused to the upstream regulatory ABI4 sequences at different stages of plant development. Using this assay, various studies have found that under normal growing conditions, the expression of ABI4 is restricted to specific stages of development (Soderman et al., 2000; Penfield et al., 2006; Bossi et al., 2009; Cui et al., 2012). Transcription of *ABI4* is detected in the embryo during most stages of seed development, but its expression is undetectable in dry seeds. Shortly after seed imbibition (24 h), the *ABI4* transcript accumulates again in most of the germinating seedling, and its expression continues through the first days of the seedling emergence. In these stages, *ABI4* is expressed in the cotyledons, the hypocotyls, and the root tips, but it is barely detectable in the true leaves (Soderman et al., 2000; Bossi et al., 2009; Cui et al., 2012). The expression of *ABI4* in later developmental stages was reported only in the anthers (Soderman et al., 2000). However, the lack of ABI4 expression in later plant developmental stages is difficult to reconcile with the participation of this factor during processes that operate in later developmental stages, such as lateral root development or the retrograde regulation responses expected to be active in true leaves. Two recent studies have reported the expression of *ABI4* in the vascular tissue of true leaves, phloem, companion cells and parenchyma of 11-day-old roots (Shkolnik-Inbar and Bar-Zvi, 2010, 2011). The differences in these expression patterns may reflect the promoter fragments used in each case: 3 or 2.6 kb in the initial studies versus 2 kb in the more recent ones. A possible explanation of these results is that some regulatory elements required for tissue-specific regulation (repressors) were excluded in the 2 kb fragment. Available data support this possibility; in the glucose-insensitive mutant *gin6*, the defects associated with altered *ABI4* expression in normal conditions or in the presence of sugars are a direct consequence of a T-DNA insertion 2 kb upstream of the ABI4 translation initiation site (Arenas-Huertero et al., 2000). Additionally, a recent analysis demonstrated that the SCARECROW (SCR) regulator represses the expression of *ABI4* through direct binding to the ABI4 promoter between -2.3 and -2.6 kb from its transcription initiation site (Cui et al., 2012). These results demonstrate that ABI4 can be expressed in later developmental stages but in response to particular signals or the action of particular regulators.

## FACTORS THAT REGULATE ABI4

The expression of *ABI4* is regulated by various factors, including ABI4 itself, which is an essential activator of its own expression during early seedling development (Arroyo et al., 2003; Bossi et al., 2009). As stated above, the plant-specific transcriptional regulator SCR has been found to repress *ABI4* expression in roots (Cui et al., 2012). SCR is required for the specification of the root endodermis, but recent data showed that SCR is also an important regulator in leaves (Dhondt et al., 2010). Additionally, recent evidence demonstrated that in the absence of the plant hormone ABA, the transcriptional regulator WRKY40 also represses *ABI4* expression (Shang et al., 2010). Similar to ABI4, WRKY40 has been implicated in plant defense responses, and this effect could be mediated, at least in part, by ABI4 (Shang et al., 2010; Kerchev et al., 2011). All these data demonstrate that the transcription of *ABI4* is tightly regulated by the action of different transcription factors.

A central question is whether it is possible that any of these regulators can transmit retrograde signals. One of the most interesting findings that potentially links ABI4 and retrograde chloroplast signaling is the recent identification of the transcriptional regulator PTM, which directly activates *ABI4* gene expression (Sun et al., 2011). PTM is a chloroplast membrane-bound protein that is conserved among plants that contain a homeodomain and localizes in the plastid envelope (Sun et al., 2011). Sun et al. (2011) demonstrated that in response to treatments that initiate retrograde signals such as norflurazon, lincomycin, and high light, PTM is processed by an unidentified intramembrane peptidase and released from the plastid envelope to the cytoplasm. The processed PTM accumulates in the nucleus, where it directly activates *ABI4* gene expression. Accordingly, in the *ptm* mutant, the expression of *ABI4* is reduced. Similar to ABI4, PTM is required in various retrograde signaling pathways (**Figure 3**). In the *ptm* mutant, treatments such as lincomycin and norflurazon do not repress *LHCB* expression. Interestingly, the function of PTM during *ABI4* expression appears to be related to histone modification in the ABI4 promoter, a regulatory mechanism that still has not been analyzed. This finding directly links plastid retrograde signals with the expression of *ABI4*, which in turn impacts on NEPPs. To be more confident in this conclusion, the endopeptidase responsible for PTM activation must be identified and the conditions that regulate its activity defined. It is also important to demonstrate whether *ABI4* induction mediated by PTM or other regulators alters active ABI4 protein levels because nuclear gene expression depends on the presence of an active protein.

ABI4 is tightly regulated at the post-transcriptional level (Finkelstein et al., 2011). Part of this regulation depends on the rapid turnover of the protein, which is mediated by the 26S proteasome (Finkelstein et al., 2011; Gregorio et al., unpublished data). Interestingly, ABI4 stability does not appear to be affected by ABA levels, unlike the ABI5 signaling factor (Lopez-Molina et al., 2003; Finkelstein et al., 2011). Thus, one of the major challenges in understanding the various functions of the ABI4 transcriptional regulator will be its analysis at the protein level. For example, binding of ABI4 to the CE1-like element does not appear to require additional factors, but this may not be the case with other alternative binding sites. Multifactor binding is an attractive alternative because it could provide the capacity to ABI4 to modulate NEPP in response to different signals, including the ability to affect different retrograde communication pathways (Koussevitzky et al., 2007;Reeves et al., 2011). For example, overexpression of the transcription factor ASR1 (abscisic acid stress ripening 1) results in *abi* and *gin* phenotypes. These phenotypes apparently are the result of displacement of ABI4 by high ASR1 levels because the binding sites of both factors overlap in some target genes (Shkolnik and Bar-Zvi, 2008).

In addition to the well-conserved APETALA 2 (AP2) DNA-binding domain (Okamuro et al., 1997; Shigyo et al., 2006), ABI4 has other conserved regions, including a serine/threonine-rich domain of unknown function that is a putative target of phosphorylation and a putative protein–protein interaction domain at its C-terminus, likely required for transcriptional activation (Finkelstein et al., 1998; Soderman et al., 2000). Thus, ABI4 could potentially associate with other factors to modulate its own activity in response to different signals. Preliminary data using a yeast one-hybrid assay demonstrated synergisms between ABI4 and other transcription factors in the transcriptional activation of some genes (Finkelstein et al., 2011). However, until now, no interacting proteins of ABI4 have been identified. This is an important subject that merits future investigation.

In summary, although the participation of ABI4 in different retrograde signaling pathways has been observed in independent analyses, the mechanism by which this factor perceives these signals probably involves not only regulation at the expression level but also at the level of protein activity, through currently unknown post-translational regulatory mechanisms that need to be identified.

## ADDITIONAL TRANSCRIPTION FACTORS

Although ABI4 has emerged as an important integrator of various retrograde signals, there are additional factors regulating these pathways. For example, in the *abi4* mutant, the expression of *LHCB* after norflurazon or lincomycin treatment is not reduced to the level of the wild-type, but the levels are still lower than without treatment (Koussevitzky et al., 2007; Sun et al., 2011). Some factors that have been shown to participate in retrograde signaling pathways include two related proteins GLK1 and GLK2 (Waters et al., 2009). These proteins are members of the GARP superfamily (Riechmann et al., 2000) and directly activate the expression of genes involved in chlorophyll biosynthesis, light harvesting, and electron transport (Waters et al., 2009). The GLK proteins were initially identified in maize, but related proteins exist in most plants (Fitter et al., 2002; Waters et al., 2009; Wang et al., 2012). These proteins are partially redundant and are required for normal chloroplast development. The *glk1*/*glk2* double mutant in maize displays a pale green phenotype with small, partially differentiated chloroplasts. GLKs have been implicated in retrograde signaling, specifically in the plastid protein import pathway (Kakizaki et al., 2009), because in the *glk1*/*glk2* double mutant, the expression of some retrograde signaling marker genes such as *LHCB*, *RBCS*, or carbonic anhydrase (*CA1*) are less sensitive to norflurazon or lincomycin treatment than wild-type plants, resulting in a weak GUN phenotype similar to that observed in ABI4 mutant (Waters et al., 2009). This result indicates that these regulatory factors may participate in the PGE and tetrapyrrole signaling pathways. In contrast to ABI4, GLKs interact with GBFs and positively regulate the expression of NEPPs (Tamai et al., 2002). In response to alterations in plastid function (photodamage, transcription, or protein import), the level of GLKs decreases and the level of ABI4 increases, resulting in massive changes in gene expression. The participation of two independent factors might provide a more versatile regulatory mechanism (**Figure 3**).

Transcription factors that exhibit dual subcellular localization are also good candidates for components of retrograde signaling mechanisms. In addition to PTM, additional factors that exhibit dual localization have been described (Krause et al., 2012). One example is pTAC12 (HEMERA), which participates in phytochrome signaling (Chen et al., 2010) and has also been detected as part of the pTAC (Pfalz et al., 2006). It has been proposed that this factor can directly modulate the expression of plastid and nuclear genes, but this function has not yet been demonstrated. Another example is the transcriptional regulator WHIRLY1, which localizes to the plastids and the nucleus of tobacco plants (Isemer et al., 2012). In plastids, this factor is also associated with the pTAC and is translocated to the nucleus through an unknown mechanism, where it activates the expression of genes related to pathogenesis. The possible participation of these dual-localized factors in retrograde signaling responses is required to be addressed.

## CONCLUDING REMARKS

ABI4 has emerged as a central integrator of essential environmental signals such as light, carbon status, ABA, redox and organelle status, facilitating the coordination of development and central metabolic processes such as photosynthesis. Although our present understanding of the mode of action of this central regulator has advanced, there are still major questions that need to be addressed in the near future, including the impact that the transcriptional regulation of ABI4 has on the levels of its protein. An aspect which is essential to understanding the function of ABI4 as a regulatory node of different signaling pathways, including plastid retrograde signaling, is how it is regulated post-transcriptionally in addition to identifying possible interaction partners. It is still an open question whether different signals can modulate the stability or activity of ABI4 either by protein modification or through direct interaction.

After a period of research that was primarily dominated by the description of independent retrograde signaling pathways, recent findings provide compelling evidence that many of those pathways converge into common integrators. Our understanding of retrograde signaling regulation is evolving into a more dynamic process in which multiple signals are produced simultaneously. These signals can affect the expression of nuclear genes in opposing ways through the participation of convergent transcriptional factors such as ABI4. This could be a very powerful strategy to respond to subtle changes in plastid functionality. The current challenges include understanding the molecular mechanism that generates signals during plastid transcription and the molecular nature of these signals. Identification of the direct targets of GUN1 could provide important insights. Although GUN1 is the only PPR protein that has been found to be required in retrograde signaling to date, the function of other related PPRs should be evaluated.

Finally, an aspect that has to be taken into consideration is that even in the *gun1* and *abi4* mutants, NEPPs repression is not completely lost. This result supports the evidence of the existence of an independent *gun1* and *abi4* retrograde signaling pathway that modulates NEPPs expression that requires future investigation.

## Conflict of Interest Statement

The authors declare that the research was conducted in the absence of any commercial or financial relationships that could be construed as a potential conflict of interest.

## Acknowledgments

We would like to thank Dr. Virginia Walbot and Dr. Mari Salmi for their helpful comments and suggestions. This work was supported by Consejo Nacional de Ciencia y Tecnología (127546) and Dirección General de Asuntos para el Personal Académico-UNAM (IN208211-3) grants.
